# Evaluation of left cardiac chamber function with cardiac magnetic resonance and association with outcome in patients with systemic sclerosis

**DOI:** 10.1093/rheumatology/keac256

**Published:** 2022-04-28

**Authors:** Steele C Butcher, Jacqueline L Vos, Federico Fortuni, Xavier Galloo, Sophie I E Liem, Jeroen J Bax, Victoria Delgado, Madelon C Vonk, Sander I van Leuven, Miranda Snoeren, Saloua El Messaoudi, Jeska K de Vries-Bouwstra, Robin Nijveldt, Nina Ajmone Marsan

**Affiliations:** Department of Cardiology, Leiden University Medical Center, Leiden, The Netherlands; Department of Cardiology, Royal Perth Hospital, Perth, Western Australia, Australia; Cardiology, Radboud UMC, Nijmegen, The Netherlands; Department of Cardiology, Leiden University Medical Center, Leiden, The Netherlands; Department of Cardiology, San Giovanni Battista Hospital, Foligno, Italy; Department of Cardiology, Leiden University Medical Center, Leiden, The Netherlands; Department of Cardiology, Vrije Universiteit Brussel, Universitair Ziekenhuis Brussel, Brussels, Belgium; Department of Rheumatology, Leiden University Medical Center, Leiden, The Netherlands; Department of Cardiology, Leiden University Medical Center, Leiden, The Netherlands; Heart Center, University of Turku and Turku University Hospital, Turku, Finland; Department of Cardiology, Leiden University Medical Center, Leiden, The Netherlands; Heart Institute, University Hospital Germans Trias i Pujol, Badalona, Spain; Department of Rheumatology; Department of Rheumatology; Department of Radiology, Radboud University Medical Center, Nijmegen, The Netherlands; Cardiology, Radboud UMC, Nijmegen, The Netherlands; Department of Rheumatology, Leiden University Medical Center, Leiden, The Netherlands; Cardiology, Radboud UMC, Nijmegen, The Netherlands; Department of Cardiology, Leiden University Medical Center, Leiden, The Netherlands

**Keywords:** left atrial, ventricular, strain, CMR, feature-tracking, SSc

## Abstract

**Objective:**

This study aimed to determine whether lower values of feature-tracking cardiovascular magnetic resonance (CMR)-derived left atrial reservoir strain (LARS) and impaired left ventricular (LV) global longitudinal strain (GLS) were associated with the presence of symptoms and long-term prognosis in patients with SSc.

**Methods:**

A total of 100 patients {54 [interquartile range (IQR) 46–64] years, 42% male} with SSc who underwent CMR imaging at two tertiary referral centres were included. All patients underwent analysis of LARS and LV GLS using feature-tracking on CMR and were followed-up for the occurrence of all-cause mortality.

**Results:**

The median LV GLS was –21.8% and the median LARS was 36%. On multivariable logistic regression, LARS [odds ratio (OR) 0.964 per %, 95% CI 0.929, 0.998, *P* = 0.049] was independently associated with New York Heart Association (NYHA) class II–IV heart failure symptoms. Over a median follow-up of 37 (21–62) months, a total of 24 (24%) patients died. Univariable Cox regression analysis demonstrated that LARS [hazard ratio (HR) 0.94 per 1%, 95% CI 0.91, 0.97, *P* < 0.0001) and LV GLS (HR 1.10 per %, 95% CI 1.03, 1.17, *P* = 0.005) were associated with all-cause mortality, while LV ejection fraction was not. Likelihood ratio tests demonstrated that LARS provided incremental value over prognostically important clinical and imaging parameters, including late gadolinium enhancement.

**Conclusion:**

In patients with SSc, LARS was independently associated with the presence of NYHA class II–IV heart failure symptoms. Although both LARS and LV GLS were associated with all-cause mortality, only LARS provided incremental value over all evaluated variables known to be prognostically important in patients with SSc.

## Graphical Abstract

**Figure:**
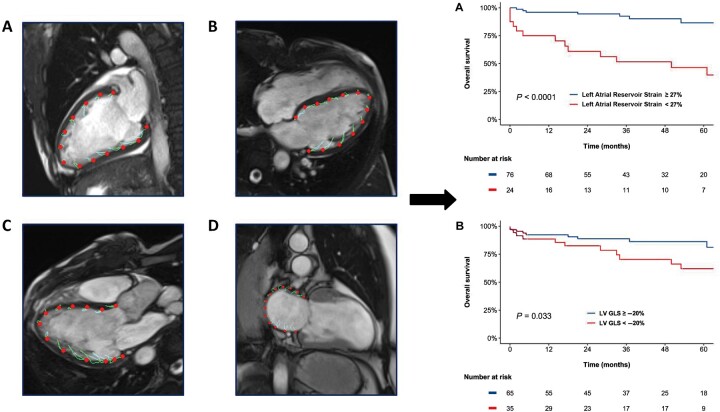

Rheumatology key messages

- Feature-tracking CMR-derived left atrial reservoir strain was independently associated with NYHA class II–IV heart failure symptoms.
- Left atrial reservoir strain provided incremental prognostic value in patients with SSc.

## Introduction

SSc is a rare autoimmune CTD characterized by multisystem microangiopathy, inflammation and fibrosis, and is associated with significant morbidity and mortality [1]. Cardiac involvement is frequent, with overt clinical manifestations evident in approximately one-quarter of patients [2]. Clinically evident cardiac involvement represents one of the main causes of death in SSc, accounting for an estimated 26% of all mortality [3]. Furthermore, with the use of more sensitive cardiac imaging techniques, subclinical cardiac disease may be detected in a significantly higher proportion of patients [2, 4–7]. Therefore, the investigation of novel imaging techniques that facilitate the early detection of cardiac involvement in patients with SSc may allow for a greater insight into disease pathophysiology and improve risk stratification.

Presently, standard evaluation of left cardiac chamber function using echocardiography and cardiovascular magnetic resonance (CMR) imaging involves only the evaluation of left ventricular (LV) ejection fraction. However, LV ejection fraction is less sensitive than LV global longitudinal strain (GLS) and may fail to detect the onset of subclinical cardiac involvement [4, 8]. Furthermore, LV diastolic dysfunction has been shown to be a key determinant of outcome in patients with SSc [9]. Left atrial strain analysis appears to provide a sensitive evaluation of LV diastolic dysfunction [8, 10], potentially providing important insights into LV function that are not possible with conventional CMR functional assessment [11].

CMR is currently indicated in patients with SSc to evaluate for evidence of inflammation and/or fibrosis, findings that may indicate primary myocardial involvement [12]. However, CMR may also be used to evaluate LV or left atrial strain with feature-tracking techniques. Compared with the evaluation of strain with echocardiography, CMR images may improve the feasibility of strain analysis by providing excellent contrast between the blood pool and myocardium, without dependence on the quality of an acoustic window [13]. However, very few studies [14, 15] have evaluated feature-tracking CMR-derived strain of the left cardiac chambers in patients with SSc, and none have examined feature-tracking CMR-derived strain of the left atrium. Moreover, no studies have provided any insight into the association between left cardiac chamber feature-tracking CMR parameters and prognosis in patients with SSc. Therefore, the present study aimed to (i) evaluate the feasibility of feature-tracking CMR-derived strain of the left atrium and left ventricle in patients with SSc, (ii) investigate the association between left cardiac chamber feature-tracking CMR-derived strain and heart failure symptoms, and (iii) investigate the association between feature-tracking CMR-derived strain of the left atrium and left ventricle and all-cause mortality.

## Methods

### Study population

The study population consisted of consecutive patients included in the prospective Leiden CCISS cohort (Combined Care In Systemic Sclerosis cohort, Leiden University Medical Center, The Netherlands) [16] and the Nijmegen SSc cohort (Radboud University Medical Center, The Netherlands) [17], who were evaluated in specifically designed health-care programs with annual organ screening. Patients were eligible for study inclusion if they were over 18 years of age, had a diagnosis of SSc and fulfilled the 2013 ACR and EULAR criteria [18], and underwent clinically indicated CMR imaging. Clinical data were collected prospectively (as part of the ongoing CCISS cohort study) or from departmental electronic health records, and included demographic characteristics, cardiovascular risk factors, modified Rodnan Skin Score (mRSS), New York Heart Association (NYHA) functional class, predicted diffusing capacity of the lungs for carbon monoxide (DLCO) and comorbidities. CMR images were analysed retrospectively for the current study. Written informed consent was waived by the local institutional review board of the Radboud University Medical Center, and written informed consent was obtained for the patients included in the CCISS cohort study at Leiden University Medical Center. The study was approved by the Institutional Review Board of Leiden University Medical Center and Radboud University Medical Center and conducted in accordance with institutional guidelines, national legal requirements, and the Declaration of Helsinki.

### Conventional CMR and echocardiographic measurements

All participants underwent CMR on 1.5-T CMR scanners, the Gyroscan ACS-NT/Intera MR system (Philips Medical Systems, Best, the Netherlands) or Siemens Avanto (Siemens Healthcare, Erlangen, Germany), or a 3.0-T CMR scanner, Ingenia MR system (Philips Medical Systems, Best, the Netherlands). All images were ECG-gated and acquired during mild end-expiration breath-holding. The scanning protocol included balanced steady-state free precession (SSFP) cine CMR images in long-axis (two-, three- and four-chamber views) and short-axis views covering the left ventricle, and late gadolinium enhancement (LGE) imaging (Supplementary Methods, available at *Rheumatology* online). LGE images were acquired 10 to 15 minutes after bolus injection of gadolinium diethylenetriamine pentaacetic acid (Magnevist, Schering, Berlin, Germany) or gadoterate meglumine (Dotarem, Guerbet, Villepinte, France) (0.15 mmol/kg) with an inversion recovery–prepared 2- or 3-dimensional segmented gradient echo pulse sequence in standard long-axis and short-axis views. Images were stored digitally for offline analysis. Right ventricular (RV) and LV end-systolic volume, end-diastolic volume, mass, and ejection fraction were measured using commercially available software (QMass Version 8.1, Medis, Leiden, The Netherlands). Left atrial volumes were measured on the cine CMR 2- and 4- chamber views, averaged, and were subsequently indexed to body surface area. The presence or absence of LGE (or hyperenhancement) was visually assessed, defined as the presence (or absence) of myocardial high signal intensity in both the short-axis and corresponding long-axis views [19].

Pulmonary artery systolic pressure (PASP) was estimated from contemporaneous transthoracic echocardiography by applying the modified Bernoulli equation to the tricuspid regurgitation jet peak velocity and adding mean right atrial pressure. Estimated right atrial pressure was calculated from the inferior vena cava diameter and its collapsibility. The presence of diastolic dysfunction was defined using guideline recommendations [20], using cut-offs of left atrial volume index specific for CMR [21, 22].

### Feature-tracking CMR-derived strain analysis

**Fig. 1:**
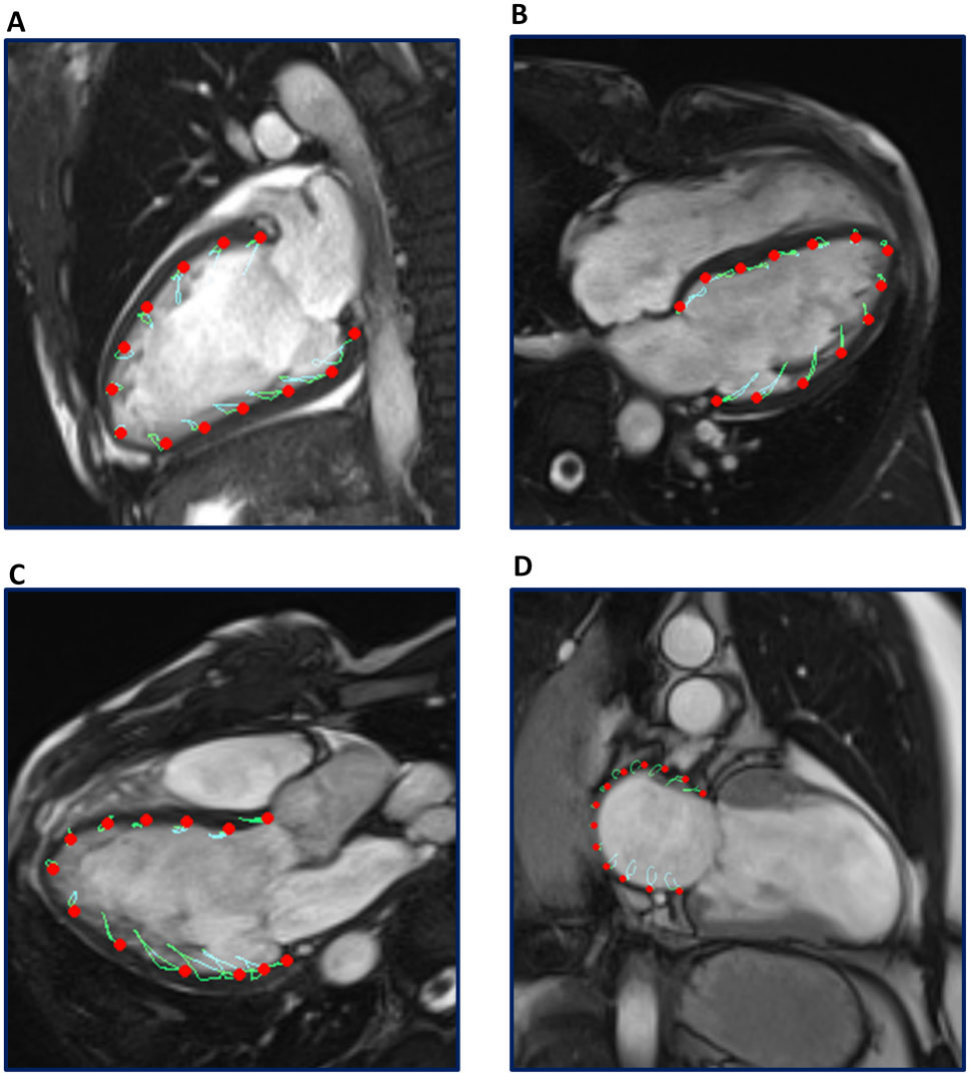
Acquisition of left atrial strain and LV GLS by feature-tracking CMR Panels A, B and C demonstrate CMR feature-tracking of the left ventricle myocardium in the 2- chamber, 4- chamber and 3- chamber long-axis cine views, respectively. LV endocardial contours were manually traced in the end-diastolic and end-systolic phase, and automatically tracked to derive an average LV GLS. Panel D demonstrates feature-tracking of the left atrial myocardium in the cine 2-chamber view. Left atrial feature-tracking was performed by manually tracing the end-diastolic and end-systolic left atrial endocardial border in the cine 2- chamber view, and LARS was estimated from the first peak of the left atrial strain curve, immediately prior to mitral valve opening. CMR: cardiac magnetic resonance; LARS: left atrial reservoir strain; LV: left ventricular; LV GLS: left ventricular global longitudinal strain.

Feature-tracking CMR-derived analysis of LV GLS was performed utilizing dedicated software (QStrain Version 2.0, Medis Medical Imaging Systems, Leiden, the Netherlands) by study site investigators who were blinded to follow-up data [23]. LV endocardial contours were manually drawn (in all three long-axis cine views) in the end-diastolic and end-systolic phase, and automatically tracked in all consecutive frames to derive an average LV GLS (Fig. 1). In addition, feature-tracking CMR-derived left atrial strain analysis was performed by manually tracing the end-diastolic and end-systolic left atrial endocardial border in the cine 2- chamber view (Fig. 1). To ensure accurate tracking of the LV and left atrial myocardium, tracking performance was visually assessed, with manual adjustments made to the contours prior to reapplication of the feature-tracking algorithm. Left atrial reservoir strain (LARS) was estimated from the first peak of the left atrial strain curve, immediately prior to mitral valve opening, using the left atrial end-systole as reference. RV strain was measured on standard 4-chamber long-axis cine images, with endocardial contours manually drawn in the end-systolic and end-diastolic phases and automatically tracked in all consecutive frames.

### Follow-up and study end points

The first end point of the study was the presence or absence of NYHA class II to IV heart failure symptoms at the time of baseline CMR. The second end point of the study was all-cause mortality at follow-up. Mortality data were collected through the Social Security Death Index or by medical record review and were complete for all patients. Follow-up began from the date of CMR, and data for all patients were included up to the last date of follow-up.

### Statistical analysis

Categorical variables are expressed as numbers and percentages, while continuous variables are presented as median and interquartile range (IQR). Differences between groups divided by the presence or absence of NYHA class II to IV heart failure symptoms were compared using the Pearson χ^2^ test for categorical variables and the Mann–Whitney U-test for continuous variables. To investigate the association of clinical and imaging parameters with the presence or absence of heart failure symptoms at baseline (defined as NYHA class ≥II), univariable logistic regression was performed. Variables with a univariable value of *P* < 0.05 were incorporated into the multivariable model. For the evaluation of the end point of all-cause mortality, penalized spline curve analysis was utilized to investigate the hazard ratio (HR) change for all-cause mortality across a range of LARS and LV GLS values. A threshold of LARS and LV GLS to dichotomize the population for Kaplan–Meier analysis was estimated using the fitted spline curves. Cumulative survival rates were calculated using the Kaplan–Meier method, and the log-rank test was used to compare groups. Associations between clinical and CMR variables and all-cause mortality were investigated by univariable Cox proportional hazards regression models. The proportional hazards assumption was verified through the assessment of scaled Schoenfeld residuals. The HR and 95% CI were calculated and reported for each variable. To test for the incremental prognostic value of LARS and LV GLS, likelihood ratio (LR) χ^2^ tests were used to assess whether feature-tracking CMR-derived strain of the left cardiac chambers added prognostic value to variables shown to be important for risk stratification in patients with SSc according to prior epidemiological data [3, 9]. Ten random patients were selected for the evaluation of intra- and inter-observer variability of LV GLS and LARS using intraclass correlation coefficients (ICCs). All tests were two-sided and *P-*values of <0.05 were considered statistically significant. Statistical analysis was performed using SPSS version 25.0 (IBM Corporation, Armonk, NY, USA) and R version 4.0.1 (R Foundation for Statistical Computing, Vienna, Austria).

## Results

### Patient characteristics

A total of 100 patients with SSc were included. The median age of the population was 54 (IQR 46 to 64) years, 42% were male and 52% had a diagnosis of dcSSc. Overlapping CTD was present in 6% of the overall population, 4% had PM overlap and 2% had SLE overlap. The median disease duration since first non-RP symptom was 38 (IQR 16 to 96) months. Patients were referred for CMR for the evaluation of cardiac involvement in 86%, screening prior to haemopoietic autologous stem-cell therapy or lung transplantation in 10%, while 4% of patients had no specific indication. The study population was dichotomized according to the presence or absence of heart failure symptoms at baseline: a total of 39 patients (39%) were in NYHA class I, while 61 (61%) were in NYHA classes II to IV. Patients in NYHA class II to IV had lower predicted DLCO than those in NYHA class I, the only significant difference between the two groups of patients. Table 1 summarizes the baseline clinical characteristics of the population.

**Table 1:** Patient clinical characteristics of the total population and divided by the presence of heart failure symptoms

Variable	Overall	NYHA class I	NYHA class II to IV	*P*-value
	(*N*=100)	(*N*=39)	(*N*=61)	
**Age, years**	54 (46–64)	52 (40–61)	56 (48–64)	0.13
**Male sex**	42 (42%)	17 (44%)	25 (41%)	0.80
**dcSSc**	50 (52%)	21 (57%)	29 (48%)	0.42
**Overlapping CTD**	6 (6.0%)	2 (5.1%)	4 (6.6%)	0.99
**mRSS**	6 (2–17)	6 (0–21)	6 (2–14)	0.87
**Disease duration since first non-RP symptom, months**	38 (16–96)	38 (16–82)	38 (16–102)	0.95
**Myositis**	19 (19%)	5 (13%)	14 (23%)	0.21
**Synovitis**	6 (6.1%)	2 (5.3%)	4 (6.7%)	0.99
**Pulmonary fibrosis^a^**	35 (35%)	11 (28%)	24 (39%)	0.25
**Predicted DLCO, %**	56 (44–68)	64 (52–77)	48 (41–61)	<0.001
**Predicted FVC, %**	88 (62–101)	96 (83–101)	80 (60–100)	0.034
**Pulmonary arterial hypertension**	6 (6.0%)	1 (2.6%)	5 (8.2%)	0.40
**Hypertension**	21 (21%)	7 (18%)	14 (23%)	0.55
**Dyslipidaemia**	10 (10%)	3 (7.7%)	7 (11%)	0.74
**Diabetes mellitus**	8 (8.0%)	1 (2.6%)	7 (11%)	0.14
**Coronary artery disease**	14 (14%)	4 (10%)	10 (16%)	0.39
**Atrial fibrillation**	15 (15%)	3 (7.7%)	12 (20%)	0.10
**COPD**	12 (12%)	3 (7.7%)	9 (15%)	0.36
**Current smoker**	15 (15%)	7 (18%)	8 (13%)	0.51
**ACEi or ARB**	42 (42%)	14 (36%)	28 (46%)	0.32
**Beta-blocker**	16 (16%)	6 (15%)	10 (16%)	0.89
**CSs**	35 (35%)	12 (31%)	23 (38%)	0.48
**CYC**	6 (6%)	2 (5.1%)	4 (6.6%)	0.99
**MTX**	12 (12%)	6 (15%)	6 (9.8%)	0.53
**AZA**	5 (5.0%)	2 (5.1%)	3 (4.9%)	0.99
**MMF**	18 (18%)	3 (7.7%)	15 (25%)	0.032
**ANA**	91 (93%)	37 (95%)	54 (92%)	0.70
**ACA**	17 (19%)	8 (22%)	9 (17%)	0.54
**Topo I**	20 (23%)	6 (18%)	14 (25%)	0.43
**CRP, mg/L**	5 (2–12)	4 (0–10)	6 (3–16)	0.081
**eGFR, mL/min/1.73 m^2^**	88 (60–90)	89 (60–90)	87 (60–90)	0.45

Median (IQR); *n* (%).

^a^ As reported on CT thorax imaging by specialist radiologists. ACEi: angiotensin-converting enzyme inhibitor, ARB: angiotensin receptor blocker, COPD: chronic obstructive pulmonary disease, DLCO: diffusing lung capacity of carbon monoxide; DM: diabetes mellitus, eGFR: estimated glomerular filtration rate, mRSS: Modified Rodnan Skin Score, NYHA: New York Heart Association, TIA: transient ischemic attack.

### CMR characteristics

The CMR characteristics of the population are presented in Table 2. The median LV end-diastolic volume was 156 (IQR 135 to 184) ml, the median left atrial volume index was 42 (IQR 34 to 54) ml/m^2^, while the LV ejection fraction was preserved (LVEF ≥50%) in the majority of the population (79%) [11]. The feasibility of analyzing LV GLS and LARS with feature-tracking CMR was excellent, with accurate measurements obtained in all 100 patients. The median LV GLS was –21.8% (IQR –24.0 to –18.1%) and the median LARS was 36% (IQR 29 to 45%). Patients with NYHA class II to IV heart failure symptoms had lower values of LARS compared with those in NYHA class I, while there was no difference in LV ejection fraction, LV GLS, cardiac index, presence of LGE or left atrial volume index between groups. Imaging characteristics divided according to the presence or absence of specific antibodies associated with SSc, systemic hypertension and coronary artery disease are displayed in Supplementary Tables S1–S4, respectively (all available at *Rheumatology* online). Clinical and imaging parameters associated with LARS are presented in Supplementary Table S5, available at *Rheumatology* online. The ICCs for intra- and inter-observer variability for LV GLS were 0.988 and 0.967, and for LARS, 0.963 and 0.917, demonstrating excellent agreement (Supplementary Table S6, available at *Rheumatology* online).

**Table 2:** Imaging characteristics of the total population and divided by the presence of heart failure symptoms

Variable	Overall	NYHA class I	NYHA class II to IV	*P*-value
	(*N*=100)	(*N*=39)	(*N*=61)	
**CMR parameters**				
**Heart rate, bpm**	73 (65–83)	71 (63–81)	76 (67–86)	0.068
**LV end-diastolic volume, ml**	156 (135–184)	170 (140–197)	152 (133–178)	0.14
**LV end-systolic volume, ml**	64 (49–85)	65 (52–84)	63 (45–87)	0.62
**LV ejection fraction, %**	60 (51–66)	61 (53–65)	58 (50–66)	0.55
**LV mass index, g/m^2^**	58 (49–66)	61 (52–69)	56 (47–65)	0.093
**LV GLS, %**	–21.8 (–24.0 to –18.1)	–22.6 (–24.5 to –19.5)	–20.9 (–23.8 to –17.5)	0.14
**Left atrial volume index, ml/m^2^**	42 (34–54)	42 (35–50)	41 (34–56)	0.98
**LARS, %**	36 (29–45)	41 (34–47)	35 (25–41)	0.004
**Pericardial effusion**	22 (23%)	8 (21%)	14 (25%)	0.66
**Presence of late gadolinium enhancement**	20 (21%)	6 (15%)	14 (25%)	0.26
**RV end-diastolic volume, ml**	154 (129–185)	155 (129–193)	153 (129–179)	0.64
**RV end-systolic volume, ml**	74 (55–94)	76 (55–93)	74 (57–94)	0.59
**RV ejection fraction, %**	52 (47–59)	55 (49–61)	51 (45–57)	0.051
**RV mass, g**	20 (16–25)	22 (16–28)	19 (14–23)	0.078
**RV GLS, %**	–27 (–31 to –22)	–28 (–32 to –23)	–26 (–31 to –21)	0.23
**RA volume index, ml/m^2^**	45 (33–58)	44 (35–50)	47 (33–60)	0.12
**Echocardiographic parameters**				
**Diastolic function**				0.16
Normal diastolic function	56 (59%)	26 (68%)	30 (53%)	
Indeterminate diastolic function	14 (15%)	6 (16%)	8 (14%)	
Diastolic dysfunction	25 (26%)	6 (16%)	19 (33%)	
**PASP, mmHg**	28 (22–35)	27 (23–31)	30 (22–39)	0.30

Median (IQR); *n* (%). CMR: cardiac magnetic resonance; IQR: interquartile range; LARS: left atrial reservoir strain; LV: left ventricular; GLS: global longitudinal strain; PASP: pulmonary artery systolic pressure; RA: right atrial; RV: right ventricular; TR: tricuspid regurgitation.

### Association between feature-tracking CMR derived strain and symptoms

To investigate the association between NYHA class II to IV heart failure symptoms and feature-tracking CMR parameters, univariable logistic regression analyses were performed (Table 3). On univariable analysis, only predicted DLCO, predicted forced vital capacity (FVC) and LARS (Supplementary Fig. S1, available at *Rheumatology* online) were associated with the presence of NYHA class II to IV heart failure symptoms at baseline. Notably, LV GLS, LV ejection fraction, LGE and left atrial volume index did not demonstrate an association on univariable analysis. On multivariable analysis, LARS (OR 0.964 per %, 95% CI 0.929, 0.998, *P* = 0.049) and predicted DLCO (OR 0.957 per %, 95% CI 0.926, 0.988, *P* = 0.008) retained an independent association with the presence of NYHA class II to IV heart failure symptoms at baseline.

**Table 3:** Univariable and multivariable logistic regression for NYHA class II–IV symptoms

	Univariable			Multivariable		
**Variable**	**OR**	**95% CI**	***P*-value**	**OR**	**95% CI**	***P*-value**
**Age, years**	1.03	1.00, 1.06	0.079			
**Male sex**	0.90	0.40, 2.04	0.80			
**Current smoker**	0.69	0.23, 2.14	0.51			
**Atrial fibrillation**	2.94	0.86, 13.6	0.11			
**Diffuse SSc**	0.71	0.31, 1.62	0.42			
**COPD**	2.08	0.57, 9.84	0.30			
**Pulmonary arterial hypertension**	3.39	0.52, 66.4	0.27			
**Predicted DLCO, %**	0.95	0.92, 0.98	<0.001	0.96	0.93, 0.99	0.008
**Predicted FVC, %**	0.98	0.96, 1.00	0.023	0.99	0.97, 1.02	0.63
**Pulmonary fibrosis**	1.65	0.70, 4.03	0.26			
**Diastolic function**			0.17			
Normal diastolic function	*–*	*–*				
Indeterminate diastolic function	1.16	0.36, 3.77	0.81			
Diastolic dysfunction	2.74	0.95, 7.90	0.061			
**PASP, mmHg**	1.04	1.00, 1.09	0.12			
**LV ejection fraction, %**	0.98	0.94, 1.02	0.29			
**RV ejection fraction, %**	0.97	0.92, 1.01	0.13			
**LV mass index, g/m^2^**	0.98	0.95, 1.01	0.18			
**LV GLS, %**	1.08	1.00, 1.17	0.078			
**LA volume index, ml/m^2^**	1.01	0.99, 1.03	0.48			
**LARS, %**	0.95	0.92, 0.98	0.004	0.96	0.93, 1.00	0.049
**Late gadolinium enhancement**	1.83	0.66, 5.65	0.26			

COPD: chronic obstructive pulmonary disease; DLCO: diffusing capacity for carbon monoxide; FVC: forced vital capacity; GLS: global longitudinal strain; LARS=left atrial reservoir strain; LV: left ventricular; OR: odds ratio.

### Association between feature-tracking CMR-derived strain and outcome

**Fig. 2:**
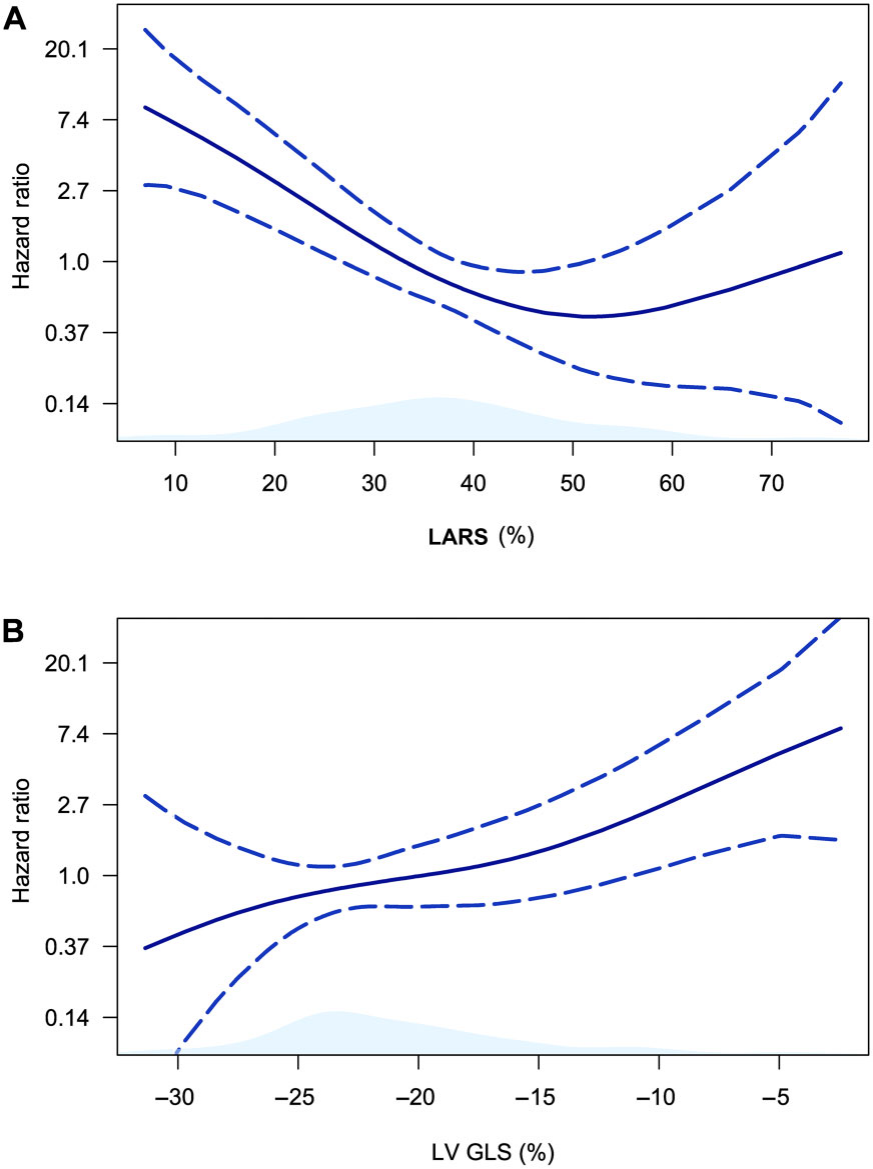
Spline curves demonstrating the hazard ratio for all-cause mortality according to LARS and LV GLS The curves in (A) and (B) demonstrate the hazard ratio change for all-cause mortality with 95% CIs (blue dotted lines) in patients with SSc, across a range of values of LARS (A) and LV GLS (B) at the time of CMR. CMR: cardiac magnetic resonance; LARS: left atrial reservoir strain; LV GLS: left ventricular global longitudinal strain.

**Fig. 3:**
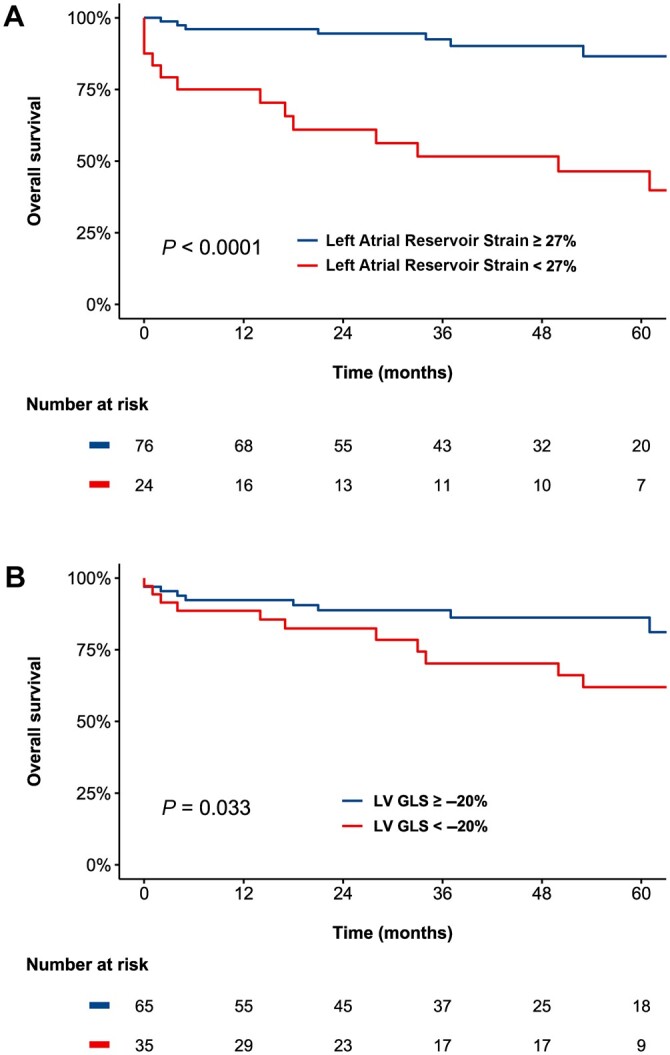
Kaplan–Meier curves for all-cause mortality for parameters of CMR feature-tracking for patients with SSc Panel A demonstrates the Kaplan–Meier curve for LARS derived by feature-tracking CMR at a cut-off of LARS of 27%, while Panel B shows the Kaplan–Meier curve for LV GLS derived by feature-tracking CMR at a cut-off of –20%. CMR: cardiac magnetic resonance; LARS: left atrial reservoir strain; LV GLS: left ventricular global longitudinal strain.

Over a median follow-up of 37 (IQR 21 to 62) months, a total of 24 (24%) patients died. Spline curve analyses were performed to investigate the association between values of LARS (Fig. 2A), LV GLS (Fig. 2B) and all-cause mortality. These analyses demonstrated significant increases in the HR for all-cause mortality, with progressively lower values of LARS and impaired LV GLS. Subsequently, to dichotomize the population for Kaplan–Meier analyses, cut-offs of 27% for LARS and –20% for LV GLS were estimated from the respective spline curves. Kaplan–Meier analysis demonstrated significantly worse survival for patients with LARS of <27% when compared with patients with a LARS of ≥27% (75% and 46% *vs* 96% and 87%, at 1 and 5 years of follow-up, respectively, *P* < 0.0001, Fig. 3A). Likewise, patients with an LV GLS of <–20% had significantly worse survival than those with an LV GLS of ≥–20% (89% and 62% *vs* 92% and 86%, at 1 and 5 years of follow-up, respectively, *P* = 0.033, Fig. 3B). Univariable Cox regression analysis demonstrated an association between all-cause mortality and LARS (HR 0.94 per %, 95% CI 0.91, 0.97, *P* < 0.0001), and all-cause mortality and LV GLS (HR 1.10 per %, 95% CI 1.03, 1.17, *P* = 0.005). In addition, an association was observed between all-cause mortality and LGE, PASP, atrial fibrillation, diastolic dysfunction, male sex, NYHA functional class, and LA volume index, although not with LV ejection fraction (Supplementary Table S7, available at *Rheumatology* online).

### Incremental prognostic value of feature-tracking CMR-derived strain

To evaluate the incremental prognostic value of LARS for the end point of all-cause mortality, LR tests were performed. LARS demonstrated incremental value over the presence of LGE (LR χ^2^ 7.82, *P* = 0.005), atrial fibrillation (LR χ^2^ 9.24, *P* = 0.002), LV diastolic dysfunction (LR χ^2^ 4.59, *P* = 0.032), DLCO (LR χ^2^ 9.95, *P* = 0.002), predicted FVC (LR χ^2^ 8.26, *P* = 0.004), PASP (LR χ^2^ 5.11, *P* = 0.024), RV ejection fraction (LR χ^2^ 9.71, *P* = 0.002), NYHA class II to IV heart failure symptoms (LR χ^2^ 8.49, *P* = 0.004) and left atrial volume index (LR χ^2^ 7.84, *P* = 0.005). Likewise, LR tests were utilized to evaluate the incremental prognostic value of LV GLS. LR testing demonstrated that LV GLS provided incremental prognostic value over atrial fibrillation (LR χ^2^ 6.24, *P* = 0.013), RV ejection fraction (LR χ^2^ 4.73, *P* = 0.030), NYHA class II to IV heart failure symptoms (LR χ^2^ 5.52, *P* = 0.019), DLCO (LR χ^2^ 5.41, *P* = 0.020), predicted FVC (LR χ^2^ 3.92, *P* = 0.048) and LA volume index (LR χ^2^ 4.68, *P* = 0.030), although it was not incremental over the presence of LGE (LR χ^2^ 3.21, *P* = 0.073), PASP (LR χ^2^ 2.33, *P* = 0.13) or LV diastolic dysfunction (LR χ^2^ 1.10, *P* = 0.29). Moreover, LARS demonstrated significant incremental prognostic value over a model including LV GLS (LR χ^2^ 6.42, *P* = 0.011), while LV GLS did not provide additional prognostic information to a model including LARS (LR χ^2^ 0.60, *P* = 0.44). Likewise, evaluation of LV diastolic dysfunction did not provide additional prognostic value to a model including LARS (LR χ^2^ 3.50, *P* = 0.17).

## Discussion

From the analysis of this large cohort of patients with SSc undergoing CMR, the major findings were as follows: (i) LARS derived by CMR was independently associated with the presence of heart failure symptoms at baseline, and (ii) LARS and LV GLS were associated with all-cause mortality, although only LARS provided incremental value over all included variables known to be prognostically important in patients with SSc.

### Association between feature-tracking CMR-derived LARS and NYHA functional class

Prior literature has demonstrated an independent relationship between NYHA functional class and all-cause mortality in patients with SSc [3, 24, 25]. However, until now, the association between imaging parameters of cardiac structure and/or function and NYHA functional class had not been investigated. In the present study, an independent association between NYHA class II to IV heart failure symptoms and feature-tracking CMR-derived LARS and predicted DLCO was observed. In contrast, no significant association was observed between NYHA functional class and LV ejection fraction, LV GLS or LV diastolic dysfunction. While the association with predicted DLCO was likely due to direct pulmonary involvement (i.e. interstitial lung disease and/or pulmonary arterial hypertension), the association between NYHA functional class and LARS may reflect early primary myocardial involvement and LV diastolic dysfunction. In addition, lower values of LARS may suggest left heart disease as a contributing etiology of heart failure symptoms and elevated pulmonary pressures in patients with SSc, necessitating careful evaluation with right heart catheterization. In SSc, microangiopathy and fibroblast dysfunction may lead to LV diastolic dysfunction, left atrial dysfunction, left atrial fibrosis and eventually, elevated filling pressures [1, 2]. Left atrial dysfunction is associated with the development of atrial fibrillation and may increase the risk of pulmonary edema and pulmonary hypertension in the setting of elevated filling pressures [26, 27]. In addition, left atrial dysfunction may represent an earlier stage of left atrial remodelling compared with left atrial dilation, and for patients with SSc, may provide a more sensitive evaluation of LV diastolic dysfunction compared with conventional echocardiographic criteria [8, 10, 28, 29]. Indeed, most patients in the present study had a normal LV ejection fraction (≥50%), a clinical setting where standard evaluation of diastolic dysfunction may produce an indeterminate result and where LARS analysis may be particularly useful.

### Association between left cardiac chamber function on CMR and all-cause mortality

The present study is the largest to evaluate feature-tracking CMR-derived strain of the left ventricle in patients with SSc, and the only study to evaluate feature-tracking CMR-derived strain of the left atrium [14, 15]. In addition, it is the first study in patients with SSc to investigate the association between CMR feature-tracking of the left atrium and left ventricle and outcome. Nonetheless, an association between feature-tracking CMR-derived strain of the left ventricle and all-cause mortality has been demonstrated in other heart failure populations, such as patients with preserved ejection fraction [30], non-ischaemic cardiomyopathy and ischaemic cardiomyopathy [23]. Likewise, an association between feature-tracking CMR-derived strain of the left atrium and mortality has been demonstrated in patients with hypertrophic cardiomyopathy [31] and with myocardial infarction [32]. The association observed between feature-tracking CMR-derived LARS and all-cause mortality following adjustment for important confounding variables (including NYHA functional class, DLCO, LVEF, left atrial volume index, LV diastolic dysfunction and PASP), suggests that this parameter may be particularly useful for risk stratification in patients with SSc. Indeed, although LV diastolic dysfunction has been shown to be a key determinant of mortality in SSc, the present study demonstrates that feature-tracking CMR-derived LARS provides incremental prognostic value to a model including LV diastolic dysfunction, while LV diastolic dysfunction does not provide incremental prognostic information to a model including LARS [9]. This may be because feature-tracking CMR-derived LARS provides a more sensitive evaluation of diastolic dysfunction than conventional echocardiographic methods, detecting early functional cardiac involvement [8]. In addition, it is possible that LARS demonstrated significant prognostic value over LV GLS because diastolic dysfunction may precede and be prognostically more important than systolic dysfunction (even if subtle or subclinical) in patients with SSc [9, 33]. Further large-scale prospective studies are required to confirm the clinical utility of feature-tracking CMR-derived strain of the left atrium for the risk stratification of patients with SSc.

### Clinical implications

While echocardiography remains the first investigation of choice for the evaluation of cardiac structure and/or function in patients with SSc, CMR represents the gold standard for chamber quantification and the detection of subclinical disease [12]. In routine clinical practice, the evaluation of feature-tracking CMR-derived strain could be performed to provide an additional prognostic tool in patients where further tissue characterization or cardiac chamber quantification was already indicated. In addition, CMR provides excellent feasibility for the evaluation of left atrial and LV strain. Existing literature suggests that the feasibility of the evaluation of left atrial and LV strain with speckle-tracking echocardiography is approximately 85 to 95% [34] and 80% [35], respectively. This is in contrast to the feasibility of 100% observed in the present study. However, this comparison of feasibility is indirect, and further prospective studies are required to evaluate differences in feasibility between CMR feature-tracking strain and speckle-tracking echocardiography. In addition, because CMR feature-tracking strain is derived from standard cine SSFP views, assessment can be seamlessly integrated into the standard CMR evaluation of patients with SSc, with accurate evaluation typically performed in less than 5 minutes per patient.

### Limitations

This study is limited by a retrospective, observational design, and patient enrollment from two specialist academic referral centres may create a selection bias. Moreover, CMR data were not acquired for the explicit purpose of evaluating left cardiac chamber function; rather, CMR was performed as a component of routine clinical practice. In addition, this study was not designed to evaluate whether left cardiac chamber strain assessed by CMR offered incremental prognostic value over left cardiac chamber strain evaluated by speckle-tracking echocardiography. Further studies adequately powered to detect any differences in prognostic utility are required to evaluate this question. Furthermore, data regarding the cause of death were not available. For the end point of all-cause mortality, multivariable analysis adjusting for an extensive number of covariates in a single model was not feasible due to the limited number of events and the risk of model overfitting. In addition, due to missing data and CMR imaging protocols/machines varying between institutions, analyses of T1 imaging data were not performed. The prevalence of pulmonary arterial hypertension was also likely underestimated, as right heart catheterization was not performed for all patients.

## Conclusion

In patients with SSc, LARS was independently associated with the presence of NYHA class II to IV heart failure symptoms at baseline. In addition, although both LARS and LV GLS were associated with all-cause mortality, only LARS provided incremental value over variables known to be prognostically important in patients with SSc.

*Funding:* This work was supported by funding from the European Society of Cardiology (ESC Research Grant App000080404) to S.C.B.

*Disclosure statement:* The Department of Cardiology of the Leiden University Medical Center received research grants from Abbott Vascular, Bioventrix, Medtronic, Biotronik, Boston Scientific, GE Healthcare and Edwards Lifesciences. J.J.B. received speaking fees from Abbott Vascular. N.A.M. received speaking fees from GE Healthcare and Abbott Vascular and is in the Medical Advisory Board of Philips Ultrasound. V.D. received speaker fees from Abbott Vascular, Medtronic, MSD, Novartis, Edwards Lifesciences and GE Healthcare. R.N. received speaker fees from Sanofi Genzyme and Bayer, and has received research grants from Philips and Biotronik. M.C.V. received research grants from Boehringer Ingelheim, Ferrer, Galapagos, and Janssen, and consulting fees from Boehringer Ingelheim, Corbus, and Janssen. The remaining authors have nothing to disclose.

## Supplementary Material

keac256_Supplementary_Data

## Data Availability

The data that support these findings are available on reasonable request to the corresponding author.
