# Supplementary material for: Evaluation of left cardiac chamber function with cardiac magnetic resonance and association with outcome in patients with systemic sclerosis
Source: Rheumatology (Oxford). 2022 Apr 28;62(SI):SI20–31. doi: 10.1093/rheumatology/keac256 (PMC9910570; doi:10.1093/rheumatology/keac256)
Supplement: keac256_Supplementary_Data [file keac256_supplementary_data.docx]

***SUPPLEMENTARY MATERIAL***

**Evaluation of Left Cardiac Chamber Function with Cardiac Magnetic Resonance and Association with Outcome in Patients with Systemic Sclerosis**

**Authors:** Steele C. Butcher*^1,2^, MD, MPhil; Jacqueline L. Vos*^3^, MD; Federico Fortuni, MD^1,4^; Xavier Galloo^1,5^, MD; Sophie I.E. Liem^6^, MD; Jeroen J Bax^1,7^, MD, PhD; Victoria Delgado^1,8^, MD, PhD; Madelon C. Vonk^9^, MD, PhD; Sander I. van Leuven^9^, MD, PhD; Miranda Snoeren^10^, MD; Saloua El Messaoudi^3^, MD, PhD; Jeska K. de Vries-Bouwstra^6^, MD, PhD; Robin Nijveldt^3^, MD, PhD; Nina Ajmone Marsan^1^, MD, PhD.

1. Department of Cardiology, Leiden University Medical Center, 2300RC Leiden, The Netherlands.
2. Department of Cardiology, Royal Perth Hospital, Perth, Western Australia, Australia.
3. Cardiology, Radboud UMC, Nijmegen, The Netherlands
4. Department of Cardiology, San Giovanni Battista Hospital, Foligno, Italy
5. Department of Cardiology, Vrije Universiteit Brussel, Universitair Ziekenhuis Brussel, Brussels, Belgium
6. Department of Rheumatology, Leiden University Medical Center, The Netherlands
7. Heart Center, University of Turku and Turku University Hospital, Turku, Finland
8. Heart Institute, University Hospital Germans Trias i Pujol, Badalona, Spain
9. Department of Rheumatology, Radboud University Medical Center, Nijmegen, The Netherlands
10. Department of Radiology, Radboud University Medical Center, Nijmegen, The Netherlands

*These authors contributed equally to this work.

**Methods (Supplementary):**

**Conventional CMR and echocardiographic measurements**

All participants underwent CMR on 1.5-T CMR scanners, the Gyroscan ACS-NT/Intera MR system (Philips Medical Systems, Best, the Netherlands) or Siemens Avanto (Siemens Healthcare, Erlangen, Germany), or a 3.0-T CMR scanner, Ingenia MR system (Philips Medical Systems, Best, the Netherlands). All images were ECG-gated and acquired during mild end-expiration breath-holding. The scanning protocol included balanced steady-state free precession (SSFP) cine CMR images in long-axis (two-, three- and four-chamber views) and short-axis views covering the left ventricle, and late gadolinium enhancement (LGE) imaging. For the 1.5-T Gyroscan ACS-NT/Intera MR system, typical imaging parameters were: field of view 400×320 mm, matrix 256×206 pixels, slice thickness 10 mm (no slice gap), flip angle 35°, echo time 1.67 ms and repetition time 3.3 ms. For the 1.5-T Siemens Avanto MR system the typical imaging parameters were: field of view 340x340 mm (ranging 300-380 and 244-380), matrix 256x256 pixels (range: 208-256), slice thickness of 5mm (range 5-8mm) and slice gap of 5 (between 4-5mm), flip angle 75-80°, echo time 1.5-1.8 ms, and a temporal resolution of <50ms. For the 3.0-T Ingenia MR system typical parameters were as follows: field of view 400×350 mm, matrix 232×192 pixels, slice thickness 8 mm (no slice gap), flip angle 45°, echo time 1.5 ms and repetition time 3.0 ms. LGE images were acquired 10 to 15 minutes after bolus injection of gadolinium diethylenetriamine pentaacetic acid (Magnevist, Schering, Berlin, Germany) or gadoterate meglumine (Dotarem, Guerbet, Villepinte, France) (0.15mmol/kg) with an inversion recovery 2- or 3-dimensional segmented inversion recovery prepared gradient echo pulse sequence in standard long-axis and short-axis views. Images were stored digitally for offline analysis. Right ventricular (RV) and LV end-systolic volume, end-diastolic volume, mass, and ejection fraction were measured using commercially available software (QMass Version 8.1, Medis, Leiden, The Netherlands). LA volumes were measured on the cine CMR 2- and 4- chamber views, averaged, and were subsequently indexed to body surface area. The presence or absence of LGE (or hyperenhancement) was visually assessed, defined as the presence (or absence) of myocardial high signal intensity in both the short-axis and corresponding long-axis views (19).

Pulmonary artery systolic pressure (PASP) was estimated from contemporaneous transthoracic echocardiography by applying the modified Bernoulli equation to the tricuspid regurgitation jet peak velocity and adding mean right atrial pressure. Estimated right atrial pressure was calculated from the inferior vena cava diameter and its collapsibility. The presence of diastolic dysfunction was defined using guideline recommendations(20), using cut-offs of LA volume index specific for CMR (21, 22).

**Supplementary Table S1: Imaging characteristics according to the presence of ANA**

| **Variable** | **Overall**  **N = 98** | **ANA Negative**  **N = 7** | **ANA Positive**  **N = 91** | **P-value** |
| --- | --- | --- | --- | --- |
| **Heart Rate, bpm** | 73 (65 – 83) | 76 (67 – 85) | 73 (65 – 82) | 0.73 |
| **LV end-diastolic volume, ml** | 156 (136 – 184) | 168 (165 – 197) | 152 (135 – 184) | 0.33 |
| **LV end-systolic volume, ml** | 64 (48 – 84) | 75 (65 – 79) | 64 (47 – 86) | 0.41 |
| **LV ejection fraction, %** | 60 (51 – 66) | 57 (53 – 63) | 60 (51 – 66) | 0.82 |
| **LV mass index, g/m^2^** | 58 (49 – 66) | 52 (48 – 63) | 58 (49 – 66) | 0.68 |
| **LV GLS, %** | -22.2 (-24.0 to -18.3) | -19.5 (-22.2 to -17.8) | -22.4 (-24.0 to -18.6) | 0.38 |
| **LA volume index, ml/m^2^** | 42 (34 – 55) | 40 (38 – 56) | 43 (34 – 54) | 0.67 |
| **LA reservoir strain, %** | 37 (29 – 45) | 41 (37 – 49) | 36 (29 – 45) | 0.36 |
| **RV end-diastolic volume, ml** | 155 (129 – 185) | 184 (179 – 197) | 152 (128 – 185) | 0.11 |
| **RV end-systolic volume, ml** | 74 (55 – 94) | 84 (77 – 87) | 71 (55 – 94) | 0.33 |
| **RV ejection fraction, %** | 52 (47 – 59) | 54 (51 – 57) | 52 (47 – 59) | 0.64 |
| **RV mass, g** | 20 (16 – 26) | 22 (18 – 27) | 20 (16 – 25) | 0.55 |
| **RV GLS, %** | -27 (-32 to -22) | -25 (-31 to -21) | -27 (-32 to -22) | 0.59 |
| **RA volume index, ml/m^2^** | 45 (35 – 58) | 49 (45 – 67) | 44 (33 – 57) | 0.25 |
| **Pericardial Effusion** | 22 (24%) | 1 (17%) | 21 (24%) | 0.99 |
| **Presence of Late Gadolinium Enhancement** | 20 (21%) | 1 (17%) | 19 (22%) | 0.99 |
| **Diastolic Function** |  |  |  | 0.13 |
| *Normal Diastolic Function* | 55 (59%) | 6 (100%) | 49 (56%) |  |
| *Indeterminate Diastolic Function* | 14 (15%) | 0 (0%) | 14 (16%) |  |
| *Diastolic Dysfunction* | 24 (26%) | 0 (0%) | 24 (28%) |  |
| Median (IQR); n (%) | | | | |
|  | | | | |

ANA = Antinuclear antibodies, CMR = Cardiac magnetic resonance; GLS = global longitudinal strain; LA = left atrial; LV = left ventricular; PASP = pulmonary artery systolic pressure, RA = right atrial, RV = right ventricular.

**Supplementary Table S2: Imaging characteristics according to the presence of ACA**

| **Variable** | **Overall**  **N = 89** | **ACA Negative**  **N = 72** | **ACA Positive**  **N = 17** | **P-value** |
| --- | --- | --- | --- | --- |
| **Heart Rate, bpm** | 73 (65 – 82) | 72 (65 – 82) | 73 (67 – 81) | 0.91 |
| **LV end-diastolic volume, ml** | 158 (137 – 186) | 164 (139 – 185) | 150 (110 – 198) | 0.52 |
| **LV end-systolic volume, ml** | 65 (48 – 86) | 67 (49 – 84) | 58 (42 – 99) | 0.56 |
| **LV ejection fraction, %** | 60 (51 – 66) | 61 (52 – 66) | 58 (52 – 63) | 0.85 |
| **LV mass index, g/m^2^** | 59 (49 – 67) | 59 (51 – 67) | 56 (46 – 66) | 0.36 |
| **LV GLS, %** | -22.0 (-24.0 – -17.9) | -22.4 (-24.2 to -17.8) | -20.0 (-23.5 to -18.5) | 0.32 |
| **LA volume index, ml/m^2^** | 43 (34 – 54) | 41 (33 – 53) | 47 (41 – 65) | 0.065 |
| **LA reservoir strain, %** | 37 (29 – 45) | 38 (29 – 47) | 33 (29 – 39) | 0.080 |
| **RV end-diastolic volume, ml** | 159 (129 – 192) | 164 (130 – 193) | 149 (107 – 177) | 0.15 |
| **RV end-systolic volume, ml** | 74 (56 – 94) | 75 (58 – 98) | 59 (49 – 89) | 0.15 |
| **RV ejection fraction, %** | 52 (47 – 59) | 52 (47 – 58) | 52 (48 – 60) | 0.51 |
| **RV mass, g** | 20.9 (16.4 – 26.1) | 21.2 (17.1 – 26.6) | 18.2 (14.7 – 24.5) | 0.26 |
| **RV GLS, %** | -27 (-32 to -22) | -27 (-32 to -22) | -26 (-31 to -19) | 0.54 |
| **RA volume index, ml/m^2^** | 45 (33 – 56) | 45 (33 – 54) | 49 (37 – 59) | 0.44 |
| **Pericardial Effusion** | 19 (23%) | 15 (23%) | 4 (24%) | >0.99 |
| **Presence of Late Gadolinium Enhancement** | 18 (21%) | 13 (19%) | 5 (31%) | 0.31 |
| **Diastolic Function** |  |  |  | 0.007 |
| *Normal Diastolic Function* | 50 (60%) | 46 (67%) | 4 (27%) |  |
| *Indeterminate Diastolic Function* | 12 (14%) | 7 (10%) | 5 (33%) |  |
| *Diastolic Dysfunction* | 22 (26%) | 16 (23%) | 6 (40%) |  |
| Median (IQR); n (%) | | | | |
|  | | | | |

ACA = Anti-centromere antibodies, CMR = Cardiac magnetic resonance; GLS = global longitudinal strain; LA = left atrial; LV = left ventricular; PASP = pulmonary artery systolic pressure, RA = right atrial, RV = right ventricular.

**Supplementary Table S3: Imaging characteristics according to the presence of systemic hypertension**

| **Variable** | **Overall**  **N = 100** | **No Systemic Hypertension**  **N = 79** | **Systemic Hypertension**  **N = 21** | **P-value** |
| --- | --- | --- | --- | --- |
| **Heart Rate, bpm** | 73 (65 – 83) | 73 (65 – 84) | 74 (67 – 79) | 0.91 |
| **LV end-diastolic volume, ml** | 156 (135 – 184) | 162 (139 – 185) | 142 (112 – 171) | 0.10 |
| **LV end-systolic volume, ml** | 64 (49 – 85) | 66 (52 – 88) | 48 (37 – 72) | 0.015 |
| **LV ejection fraction, %** | 60 (51 – 66) | 57 (49 – 65) | 62 (58 – 68) | 0.018 |
| **LV mass index, g/m^2^** | 58 (49 – 66) | 58 (49 – 66) | 54 (47 – 70) | >0.99 |
| **LV GLS, %** | -21.8 (-24.0 – -18.1) | -20.9 (-23.8 – -17.5) | -22.5 (-24.4 – -18.9) | 0.26 |
| **LA volume index, ml/m^2^** | 42 (34 – 54) | 40 (33 – 52) | 49 (38 – 61) | 0.10 |
| **LA reservoir strain, %** | 36 (29 – 45) | 37 (29 – 44) | 35 (25 – 51) | 0.80 |
| **RV end-diastolic volume, ml** | 154 (129 – 185) | 159 (132 – 191) | 148 (118 – 177) | 0.13 |
| **RV end-systolic volume, ml** | 74 (55 – 94) | 76 (56 – 95) | 59 (49 – 84) | 0.064 |
| **RV ejection fraction, %** | 52 (47 – 59) | 51 (47 – 58) | 58 (51 – 61) | 0.068 |
| **RV mass, g** | 20 (16 – 25) | 20 (15 – 26) | 19 (18 – 23) | 0.94 |
| **RV GLS, %** | -27 (-31 – -22) | -26 (-31 – -21) | -28 (-32 – -24) | 0.43 |
| **RA volume index, ml/m^2^** | 45 (33 – 58) | 44 (33 – 55) | 49 (39 – 59) | 0.21 |
| **Pericardial Effusion** | 22 (23%) | 16 (22%) | 6 (29%) | 0.56 |
| **Presence of Late Gadolinium Enhancement** | 20 (21%) | 17 (23%) | 3 (15%) | 0.55 |
| **Diastolic Function** |  |  |  | 0.68 |
| *Normal Diastolic Function* | 56 (59%) | 44 (59%) | 12 (60%) |  |
| *Indeterminate Diastolic Function* | 14 (15%) | 10 (13%) | 4 (20%) |  |
| *Diastolic Dysfunction* | 25 (26%) | 21 (28%) | 4 (20%) |  |
| Median (IQR); n (%) | | | | |
|  | | | | |

CMR = Cardiac magnetic resonance; GLS = global longitudinal strain; LA = left atrial; LV = left ventricular; PASP = pulmonary artery systolic pressure; RA = right atrial; RV = right ventricular.

**Supplementary Table S4: Imaging characteristics according to the presence of coronary artery disease**

| **Variable** | **Overall**  **N = 100** | **No CAD**  **N = 86** | **CAD**  **N = 14** | **P-value** |
| --- | --- | --- | --- | --- |
| **Heart Rate, bpm** | 73 (65 – 83) | 74 (66 – 86) | 73 (60 – 78) | 0.14 |
| **LV end-diastolic volume, ml** | 156 (135 – 184) | 156 (134 – 184) | 159 (142 – 205) | 0.57 |
| **LV end-systolic volume, ml** | 64 (49 – 85) | 64 (48 – 84) | 78 (53 – 100) | 0.23 |
| **LV ejection fraction, %** | 60 (51 – 66) | 61 (52 – 66) | 53 (42 – 60) | 0.067 |
| **LV mass index, g/m^2^** | 58 (49 – 66) | 57 (47 – 66) | 61 (55 – 66) | 0.15 |
| **LV GLS, %** | -21.8 (-24.0 – -18.1) | -22.5 (-24.0 – -18.7) | -20.5 (-21.5 – -12.7) | 0.068 |
| **LA volume index, ml/m^2^** | 42 (34 – 54) | 41 (34 – 52) | 57 (40 – 64) | 0.050 |
| **LA reservoir strain, %** | 36 (29 – 45) | 36 (29 – 45) | 36 (25 – 44) | 0.49 |
| **RV end-diastolic volume, ml** | 154 (129 – 185) | 152 (129 – 185) | 157 (124 – 207) | 0.96 |
| **RV end-systolic volume, ml** | 74 (55 – 94) | 75 (55 – 94) | 70 (59 – 112) | 0.83 |
| **RV ejection fraction, %** | 52 (47 – 59) | 52 (47 – 59) | 48 (47 – 57) | 0.54 |
| **RV mass, g** | 20 (16 – 25) | 20 (15 – 26) | 20 (18 – 22) | 0.96 |
| **RV GLS, %** | -27 (-31 – -22) | -27 (-31 – -21) | -27 (-32 – -24) | 0.55 |
| **RA volume index, ml/m^2^** | 45 (33 – 58) | 44 (33 – 54) | 57 (43 – 66) | 0.029 |
| **Pericardial Effusion** | 22 (23%) | 20 (25%) | 2 (15%) | 0.73 |
| **Presence of Late Gadolinium Enhancement** | 20 (21%) | 12 (15%) | 8 (57%) | 0.001 |
| **Diastolic Function** |  |  |  | 0.35 |
| *Normal Diastolic Function* | 56 (59%) | 51 (61%) | 5 (42%) |  |
| *Indeterminate Diastolic Function* | 14 (15%) | 11 (13%) | 3 (25%) |  |
| *Diastolic Dysfunction* | 25 (26%) | 21 (25%) | 4 (33%) |  |
| Median (IQR); n (%) | | | | |
|  | | | | |

CAD = coronary artery disease; CMR = Cardiac magnetic resonance; GLS = global longitudinal strain; LA = left atrial; LV = left ventricular; PASP = pulmonary artery systolic pressure; RA = right atrial; RV = right ventricular.

**Supplementary Table S5: Linear Regression demonstrating association between clinical and imaging characteristics with CMR feature-tracking derived LA reservoir strain**

| **Variable** | **B (95% CI)** | **P-value** |
| --- | --- | --- |
| **Age, years** | -0.11 (-0.30 to 0.08) | 0.27 |
| **Male Sex** | -2.8 (-8.4 to 2.7) | 0.32 |
| **Smoker** | 4.3 (-3.3 to 12) | 0.26 |
| **Atrial fibrillation** | -9.3 (-17 to -1.8) | 0.016 |
| **Diffuse Systemic Sclerosis** | 1.8 (-3.8 to 7.4) | 0.52 |
| **COPD** | -8.1 (-16 to 0.22) | 0.056 |
| **Predicted DLCO, %** | 0.17 (0.00 to 0.34) | 0.046 |
| **Predicted FVC, %** | 0.08 (-0.04 to 0.19) | 0.17 |
| **Pulmonary Arterial Hypertension** | -7.0 (-18 to 4.6) | 0.23 |
| **CRP** | -0.04 (-0.21 to 0.12) | 0.59 |
| **Diastolic Function** |  |  |
| *Normal Diastolic Function* | — |  |
| *Indeterminate Diastolic Function* | -9.4 (-17 to -2.1) | 0.012 |
| *Diastolic Dysfunction* | -14 (-20 to -8.1) | <0.001 |
| **LV ejection fraction, %** | 0.48 (0.28 to 0.69) | <0.001 |
| **LV mass index, g/m^2^** | -0.10 (-0.29 to 0.08) | 0.26 |
| **LV GLS, %** | -1.2 (-1.7 to -0.82) | <0.001 |
| **LA volume index, ml/m^2^** | -0.28 (-0.40 to -0.17) | <0.001 |
| **PASP, mmHg** | 0.03 (-0.21 to 0.27) | 0.81 |
| **RV ejection fraction, %** | 0.36 (0.09 to 0.62) | 0.008 |
| **RV GLS, %** | -0.58 (-1.0 to -0.19) | 0.004 |
| **Late gadolinium enhancement** | -15 (-21 to -8.3) | <0.001 |
| **Pulmonary Fibrosis** | 2.3 (-3.4 to 8.1) | 0.43 |
| **NYHA II to IV** | -8.7 (-14 to -3.3) | 0.002 |
|  | | |

CI = Confidence Interval; COPD = chronic obstructive pulmonary disease; DLCO = Diffusing capacity for carbon monoxide; GLS = global longitudinal strain; LA= left atrial; LV = left ventricular

**Supplementary Table S6: Intraobserver and interobserver variability for LV GLS and LA reservoir strain**

|  | **Intraclass Correlation Coefficient (95% CI)** |
| --- | --- |
| **Intraobserver Variability (N=10)** |  |
| LV GLS | 0.988 (0.954 to 0.997) |
| LA reservoir strain | 0.963 (0.857 to 0.991) |
| **Interobserver Variability (N=10)** |  |
| LV GLS | 0.967 (0.873 to 0.992) |
| LA reservoir strain | 0.917 (0.603 to 0.980) |

CI = Confidence Interval; GLS = global longitudinal strain; LA= left atrial; LV = left ventricular

**Supplementary Table S7: Univariable Cox Regression for All-Cause Mortality**

|  | **Univariable** | | |
| --- | --- | --- | --- |
| **Variable** | **HR** | **95% CI** | ***P*-value** |
| **Age, years** | 1.02 | 0.99 to 1.05 | 0.17 |
| **Male Sex** | 2.68 | 1.17 to 6.14 | 0.020 |
| **Current Smoker** | 1.09 | 0.32 to 3.73 | 0.88 |
| **Atrial Fibrillation** | 4.06 | 1.74 to 9.48 | 0.001 |
| **Diffuse Systemic Sclerosis** | 0.96 | 0.42 to 2.18 | 0.92 |
| **Myositis** | 1.28 | 0.48 to 3.46 | 0.62 |
| **COPD** | 1.92 | 0.71 to 5.22 | 0.20 |
| **Predicted DLCO, %** | 0.97 | 0.94 to 1.00 | 0.057 |
| **Predicted FVC, %** | 0.98 | 0.96 to 1.00 | 0.056 |
| **Pulmonary Fibrosis** | 1.32 | 0.57 to 3.07 | 0.52 |
| **NYHA Class II to IV** | 8.54 | 2.00 to 36.46 | 0.004 |
| **Pulmonary Arterial Hypertension** | 1.60 | 0.37 to 6.88 | 0.53 |
| **C-Reactive Protein, mg/L** | 1.01 | 0.99 to 1.03 | 0.20 |
| **Diastolic Function** |  |  |  |
| *Normal Diastolic Function* | — | *Reference* |  |
| *Indeterminate Diastolic Function* | 3.61 | 1.09 to 11.90 | 0.035 |
| *Diastolic Dysfunction* | 3.31 | 1.20 to 9.14 | 0.021 |
| **PASP, mmHg** | 1.03 | 1.00 to 1.06 | 0.041 |
| **LV ejection fraction, %** | 0.97 | 0.94 to 1.00 | 0.077 |
| **LV mass index, g/m^2^** | 1.01 | 0.98 to 1.03 | 0.68 |
| **Cardiac index, L/m^2^** | 0.91 | 0.58 to 1.43 | 0.69 |
| **LA volume index, ml/m^2^** | 1.02 | 1.01 to 1.04 | 0.007 |
| **Late gadolinium enhancement** | 3.42 | 1.41 to 8.27 | 0.006 |
| **RV ejection fraction, %** | 0.99 | 0.95 to 1.02 | 0.51 |
| **LA reservoir strain, %** | 0.94 | 0.91 to 0.97 | <0.001 |
| **LV GLS, %** | 1.10 | 1.03 to 1.17 | 0.005 |

CI = Confidence Interval; COPD = chronic obstructive pulmonary disease; DLCO = Diffusing capacity for carbon monoxide; FVC = forced vital capacity; GLS = global longitudinal strain; HR = hazard ratio; LA= left atrial; LV = left ventricular; NYHA = New York Heart Association; PASP = pulmonary artery systolic pressure; RV = right ventricular.

**Supplementary Figure S1: Spline curve of the probability of symptoms (NYHA II-IV) according to LA reservoir strain**


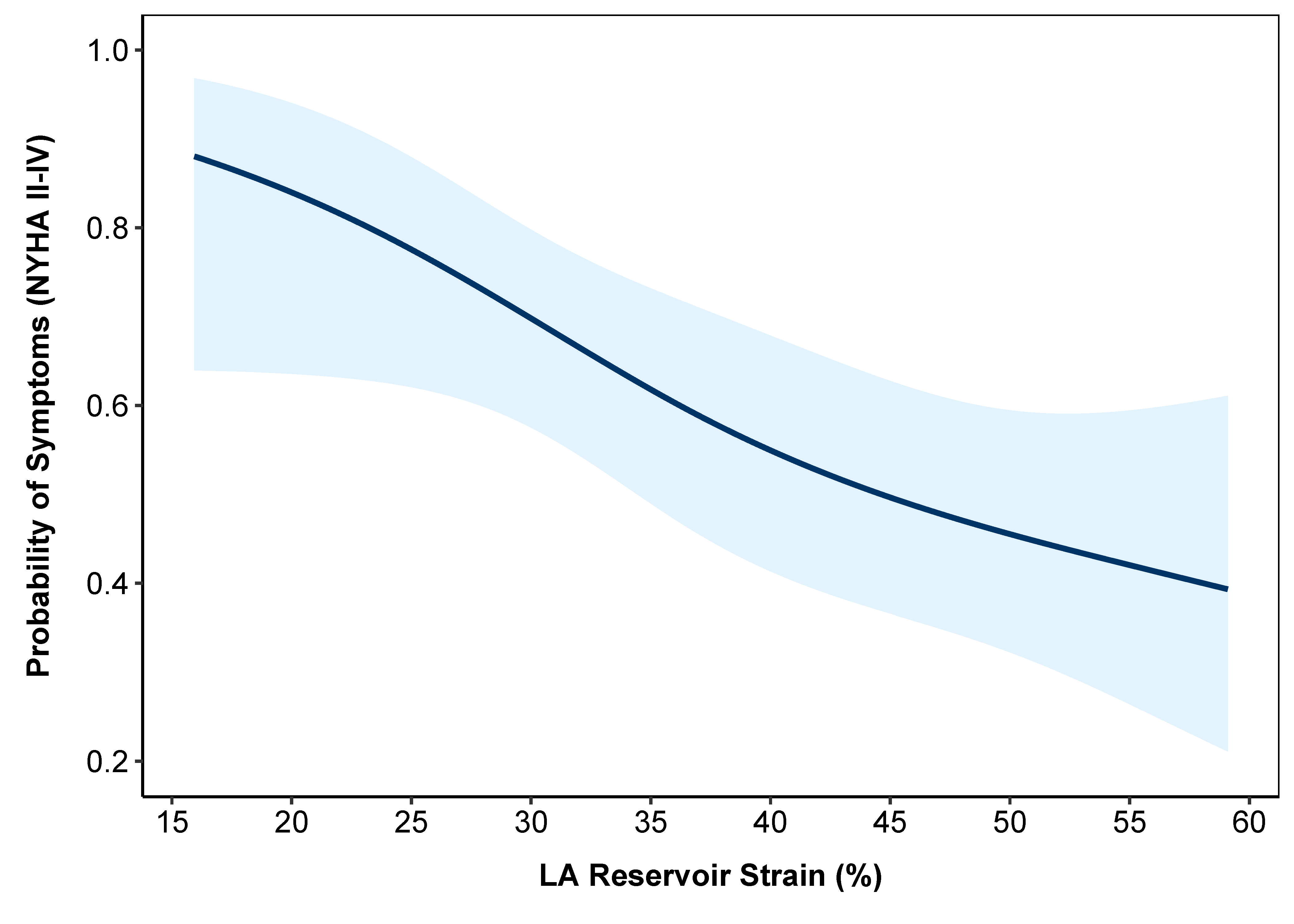


LA = left atrial; NYHA = New York Heart Association
